# Electromagnetic Sheet Forming by Uniform Pressure Using Flat Spiral Coil

**DOI:** 10.3390/ma12121963

**Published:** 2019-06-18

**Authors:** Xiaohui Cui, Dongyang Qiu, Lina Jiang, Hailiang Yu, Zhihao Du, Ang Xiao

**Affiliations:** 1 Light alloy research Institute, Central South University, Changsha 410083, China; yuhailiang@csu.edu.cn (H.Y.); dzh7695782@126.com (Z.D.); 15243678932@163.com (A.X.); 2 College of Mechanical and Electrical Engineering, Central South University, Changsha 410083, China; 15344413828@163.com; 3 State Key Laboratory of High Performance Complex Manufacturing, Central South University, Changsha 410083, China; 4 Shandong North Binhai Machinery Company, Zibo 255201, China; linajiang120583@sina.com

**Keywords:** electromagnetic sheet forming, uniform pressure, numerical simulation

## Abstract

The coil is the most important component in electromagnetic forming. Two important questions in electromagnetic forming are how to obtain the desired magnetic force distribution on the sheet and increase the service life of the coil. A uniform pressure coil is widely used in sheet embossing, bulging, and welding. However, the coil is easy to break, and the manufacturing process is complex. In this paper, a new uniform-pressure coil with a planar structure was designed. A three-dimensional (3D) finite element model was established to analyze the effect of the main process parameters on magnetic force distribution. By comparing the experimental results, it was found that the simulation results have a higher analysis precision. Based on the simulation results, the resistivity of the die, spacing between the left and right parts of the coil, relative position between coil and sheet, and sheet width significantly affect the distribution of magnetic force. Compared with the structure and magnetic force on a traditional uniform pressure coil, the planar uniform pressure coil can produce a uniform magnetic force distribution on the sheet, reduce the manufacturing difficulty, reduce manufacturing cost, and enhance the service life for the coil.

## 1. Introduction

In electromagnetic forming (EMF), the coil is a key component that converts the electrical energy stored in a capacitor into the kinetic energy required for the deformation of a workpiece. The shape of the coil determines the distribution of electromagnetic force, affecting the forming result of the workpiece. According to the deformed shape of a workpiece, the forming coil can be divided into two types: (1) The forming coil for a tube; (2) the forming coil for a flat sheet.

The tube forming coils have a solenoid structure, mainly used to achieve tube bulging and tube compression. For example, Cui et al. [1] found that electromagnetic tube bulging with multidirectional magnetic pressure can decrease the tensile stress and thickness at the easily broken position on a tube. Yu et al. [2] predicted electromagnetic tube compression and evaluated the optimum current frequency using a finite element model (FEM).

Compared with electromagnetic tube forming, electromagnetic sheet forming is more complex. Thus, there are many coil structures for sheet deformation. Cui et al. [3] accurately predicted sheet bulging using a flat spiral circular coil. Li et al. [4] obtained the biaxial tensile stress state on a sheet by using flat spiral circular coils. It was found that the forming limit of Ti–6Al–4V sheets increased by 24.37% when subjected to electromagnetic sheet bulging compared with the quasi-static condition. Cui et al. [5] used the electromagnetic incremental forming (EMIF) method to produce a large part using a small flat spiral circular coil under the condition of a small discharge energy. Cui et al. [6] placed a flat spiral circular coil at the sheet-metal end to generate radial magnetic pressure. Several steps of quasi-static stamping and magnetic-pulse forming with radial force improve the forming depth by 31% compared to quasi-static stamping. Cui et al. [7] used two flat spiral-waisted coils and a small discharge energy to manufacture a large-size part after 36 discharging and six stretching steps. Cui et al. [8] used the spiral circular coil with tower type and multidirectional magnetic pressure to reduce the corner radius of a cylindrical part. Li et al. [9] designed special specimens to obtain the uniaxial tension state by EMF using a single-turn coil. Oliveira et al. [10] designed a double spiral square coil to make sheet-free bulging and formed a flat insert cavity. However, a “dead spot” (region of low magnetic pressure) occurred on the sheet, corresponding to the center of winding of the flat spiral coil. Therefore, traditional flat actuators produce a non-uniform pressure distribution, limiting the types of parts that can be formed.

**Figure 1:**
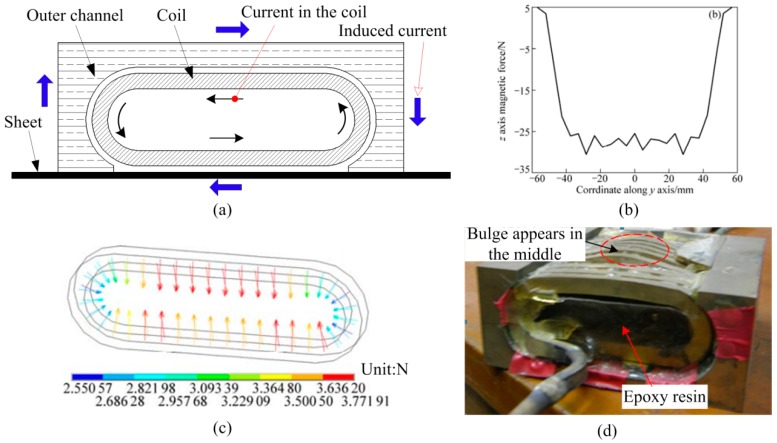
Traditional uniform-pressure coil. (**a**) Design principle, (**b**) magnetic force on sheet [17], (**c**) magnetic force on a coil [17], and (**d**) failure of a coil [18].

Thus, a new type of actuator that provided a uniform pressure distribution on a sheet was proposed by Kamal et al. [11]. Figure 1a shows the design principle of a uniform-pressure coil. The outer channel is in close contact with the sheet metal, generating the induced current on an outer channel and sheet metal composed of a closed circuit after coil discharge. Manish et al. [12] used a two-step forming process to manufacture a good phone face by using a uniform-pressure coil. Golowin et al. [13] demonstrated that the uniform pressure actuator can form a sheet metal with a complex shape and obtain fine surface features. Kinsey et al. [14] established the electromagnetic-mechanically coupled numerical model to predict the forming pressure and mechanical deformation using a uniform pressure actuator. Weddeling et al [15] proved the magnetic pulse welding is feasible and applicable by using the uniform pressure electromagnetic coil. Thibaudeau et al. [16] proposed an analytical model to design the uniform pressure coil. The designed coil can be used for electromagnetic forming and magnetic pulsed welding. Cui et al. [17] analyzed the distribution of magnetic force on a sheet and coil using a uniform pressure coil. It was found that a uniform pressure is distributed in the region corresponding to the under-surface of the coil. Thus, the coil undergoes a complex state of stress, which may cause coil failure. Figure 1d shows that a bulge appears in the bottom surface of the coil [18]. Therefore, compared to a flat spiral coil, the uniform-pressure coil in Figure 1 has two main problems: (1) The coil can easily break, and (2) the coil has a complex structure.

Therefore, a new uniform-pressure coil with a planar structure was designed in this study. A three-dimensional (3D) FEM was established to analyze the effect of the main process parameters on magnetic force distribution. Finally, the rationality of the coil design was verified according to the experiment of sheet deformation.

## 2. Design Principle of New Uniform-Pressure Coil

**Figure 2:**
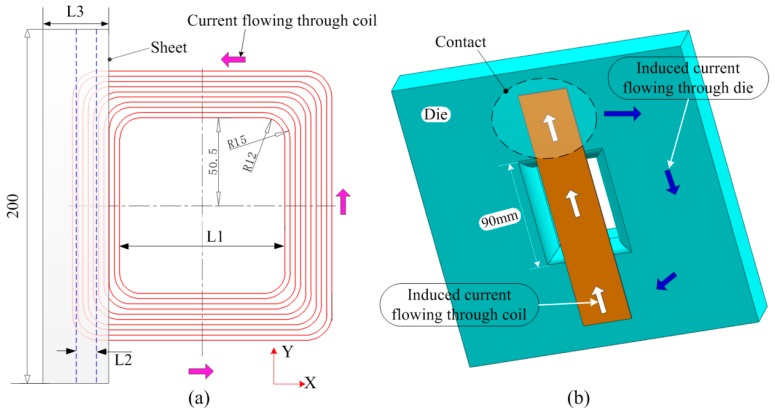
Design principle for the new uniform-pressure coil. (**a**) Coil and sheet and (**b**) induced current flowing in the sheet and die.

To obtain a uniform distribution of magnetic force on a sheet, it must be ensured that the induced current directions on the sheet are the same. Thus, the same current direction should be applied to the coil facing on the sheet region to be deformed. Based on this principle, a uniform-pressure coil with planar spiral structure was designed, as shown in Figure 2. It was assumed that the coil is loaded with a counterclockwise current. If the current in the left half of the coil flows in the negative direction of the Y-axis, the induced current on the sheet should flow in the positive direction of the Y-axis. To make the induced current constitute a closed current loop, a conductive die was set to contact with both the ends of the sheet, as shown in Figure 2b.

To analyze the distribution of magnetic force on the sheet generated by the coil, the following process parameters were analyzed: (1) The resistivity of the die; (2) the spacing between the left and right parts of the coil, set to L1; (3) the distance of the center line between the sheet and the left part of the coil, set to L2; (4) the width of the sheet, set to L3.

## 3. FEM

**Figure 3:**
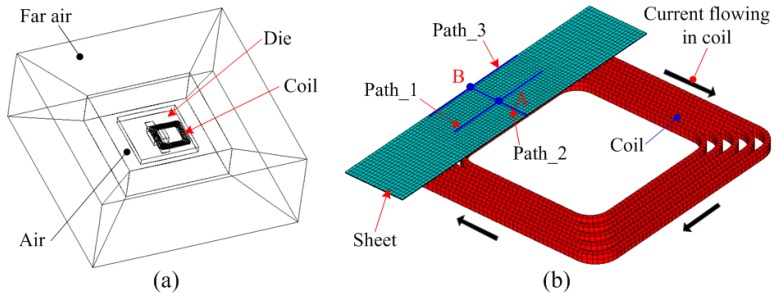
Finite-element models: (**a**) Electromagnetic field model and (**b**) coil and sheet.

In this paper, the ANSYS/EMAG software was used to calculate the magnetic force acting on the sheet, and ANSYS/LSDYNA was used to predict the sheet deformation process after coil discharge. Figure 3 shows the 3D FEM for electromagnetic forming. The electromagnetic field model consists of a far-air region, an air region, a coil, and a sheet. The element types for the far-air region, air region, coil, and sheet were infin111, solid97, solid97, and solid97, respectively. The far-field air represents the boundary conditions of the magnetic field, while the near-field air is used to transfer the magnetic field generated by the coil to both the sheet and the die. During the electromagnetic forming process, the current density was loaded on the coil. Both the sheets and the coils were divided into a hexahedral mesh. The number of nodes and elements for the sheet were 8505 and 6400, respectively. The number of nodes and elements for the coil element type were 4800 and 1600, respectively. To describe the contact between the sheet and the die in electromagnetic field analysis, the contact-region between the sheet and die was processed by common nodes. For further analysis, three paths were defined on the sheet. Path 1 and Path 2 were used to analyze the distribution of magnetic force on the sheet. Path 3 was used to describe the final deformation profile of the sheet. The intersection points of Path 1 and Path 2, as well as Path 2 and Path 3, were set to point A and B, respectively.

In this study, 0.5 mm thickness 5052-O aluminum alloy sheets were used in the following experiments. The true stress–strain for the sheet is shown in Equation (1), and the yield strength of the sheet was 105.8 MPa. The material properties of the sheet, coil, and steel die (made by Q235) are given in Table 1. To consider the effect of a high strain rate on the forming process, the properties of material were modeled using the Cowper–Symonds constitutive model as shown in Equation (2).

(1) $$\sigma_{s}=474.6{\left(\varepsilon +0.01866\right)}^{0.377}$$

(2) $$\sigma =\sigma_{s}(1+{\left(\frac{\dot{\varepsilon }}{P}\right)}^{m})$$

where σ is the dynamic flow stress, σ_s_ is quasi-static stress, *ε* is the plastic strain, $\dot{\varepsilon }$ is the strain rate, P = 6500 s^−1^, and m = 0.25 is a specific parameter of aluminum alloy.

**Figure 4:**
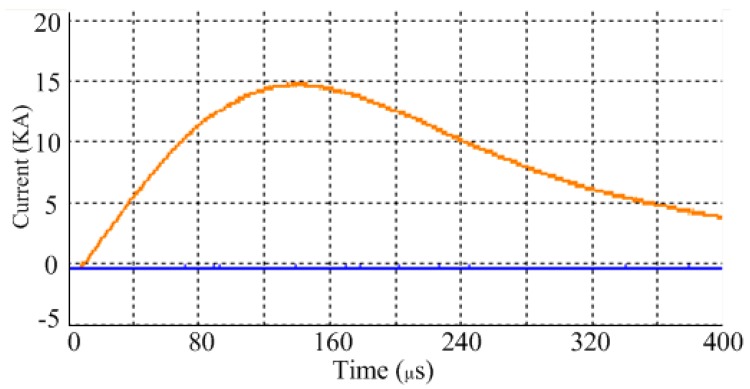
Current through the coil.

Figure 4a shows the current curves through the coil at 2.5 KV measured using a Rogowski coil. To improve the operating lifetime of capacitor, a diode and resistor were connected in parallel with the capacitor to inhibit the reverse charging of the capacitor. The current data were recorded for Δt = 1 μs and loaded into the coil to calculate the subsequent magnetic field and sheet deformation.

## 4. Effect of Process Parameters on Magnetic Force

### 4.1. Resistivity of Die

**Figure 5:**
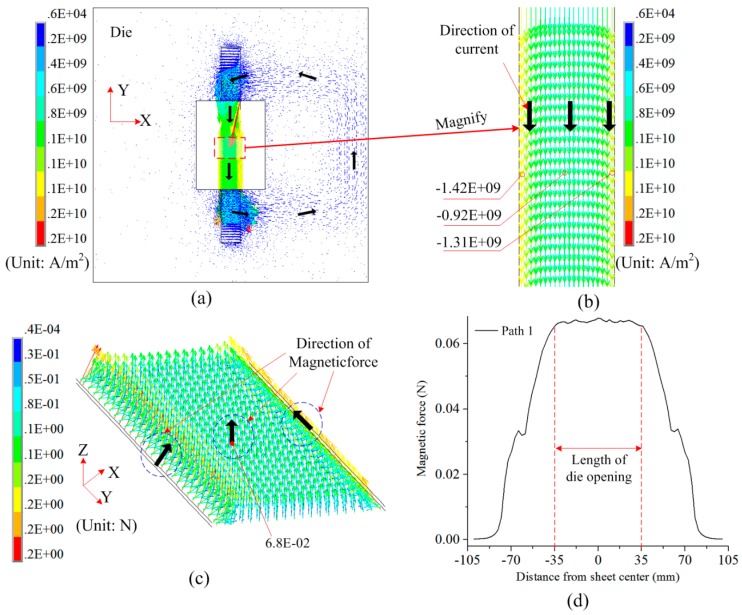
Distribution of induced current and magnetic force using the die with resistivity equal to 5e-7: (**a**) Current on the sheet and die, (**b**) current in detailed sheet region, (**c**) 3D magnetic force on the sheet, and (**d**) magnetic force along Path 1.

If L1 = 95 mm, L2 = 0 mm, and L3 = 20 mm, Figure 5 shows the induced current and magnetic force on a sheet using the die with a resistivity equal to 5e-7 Ω·m. It was found that the induced current occurs on both the sheet and die after the coil is discharged. It was assumed that the coil is loaded with clockwise current, as shown in Figure 3b. The direction of induced current on the sheet and die was opposite to the direction of current on the coil. Moreover, the induced current formed a current loop. Thus, the current with same direction generated on the sheet corresponded to the hollow region of the die. Because the width of the left half of the coil was equal to 27 mm and the width of sheet L3 was set to 20 mm, the current density in the middle of the sheet was less than those on the two sides of the sheet, as shown in Figure 5b. In addition, the current density at the right side of sheet was smaller than that at the left side of the sheet. This is because the right half of the coil can produce electromagnetic induction on the right side of the sheet. The current direction of the right half of the coil was opposite to that of left half of the coil. This will result in a lower current density at the right side of the sheet. Figure 5c shows the distribution of magnetic field force on the sheet. The direction of electromagnetic force was positive along the Z-axis as a whole, but magnetic forces existed along the X-axis at both sides of the sheet. Based on the 3D distribution of magnetic force in Figure 5c, Figure 5d shows the magnetic force along Path 1. A uniform magnetic pressure was observed at the location of −35 mm–35 mm away from the sheet center. The length, with a uniform pressure, is equal to the length of the die opening.

**Figure 6:**
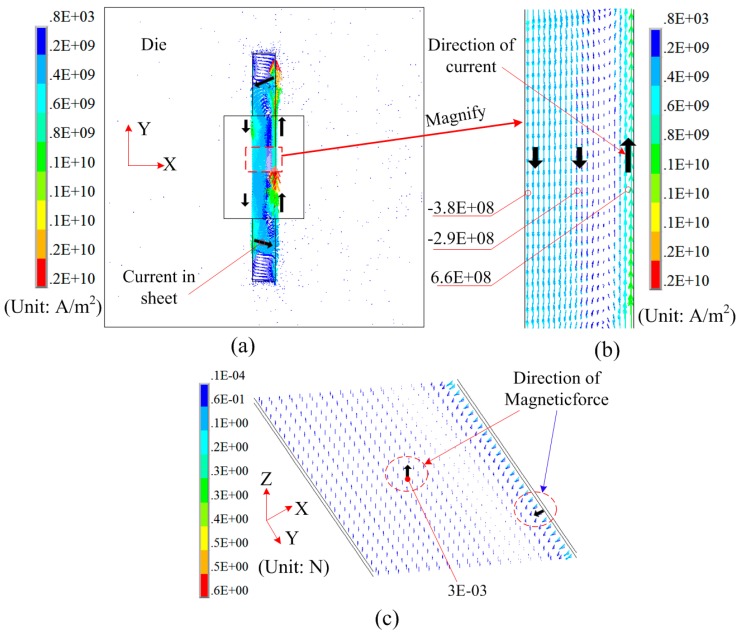
Distribution of induced current and magnetic force using the die with a resistivity of 5e-4: (**a**) Current on the sheet, (**b**) current in detailed sheet region, and (**c**) 3D magnetic force on the sheet.

If L1 = 95 mm, L2 = 0 mm, and L3 = 20 mm, Figure 6 shows the induced current and magnetic force on the sheet using the die with a resistivity of 5e-4 Ω·m. After the coil discharge, induced current can be generated on the sheet, but no induced current is generated on the die. Thus, the induced current on sheet constitutes a current loop, as shown in Figure 6a. The current density at the right side of the sheet is much larger than that at the left side of sheet, as shown in Figure 6b. Figure 6c shows the distribution of electromagnetic force on sheet. A very small magnetic force was generated on the left and middle region of the sheet. Though the magnetic force at the right side of the sheet has a large magnetic force, the direction of magnetic force is not positive along the Z-axis. Therefore, a die with a smaller resistivity should be used to generate a uniform magnetic force on the sheet according to the results shown in Figure 5 and Figure 6.

### 4.2. DistanceBetween the Left and Right Parts of Coil (L1)

**Figure 7:**
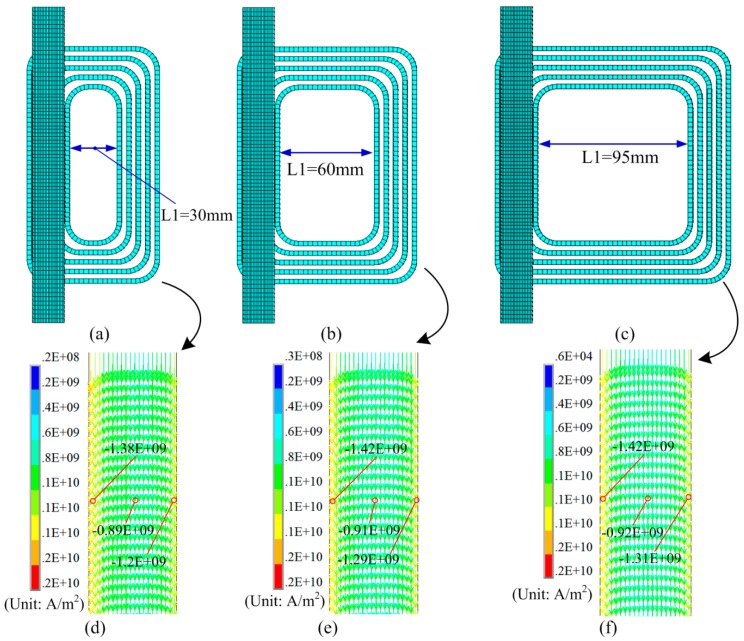
Effect of L1 on current density: (**a**) L1 = 30 mm, (**b**) L1 = 60 mm, and (**c**) L1 = 95 mm. (**d**) Current density with L1 = 30 mm, (**e**) value of current with L1 = 60 mm, and (**f**) value of current with L1 = 95 mm.

If L2 = 0 mm, L3 = 20 mm, and the resistivity of die is set to 5e-7, Figure 7 shows the effect of L1 on the current density of the sheet. With the increase in L1, the current density in the middle and side of the sheet increased. The difference in current density on the left and right sides of the sheet decreased.

**Figure 8:**
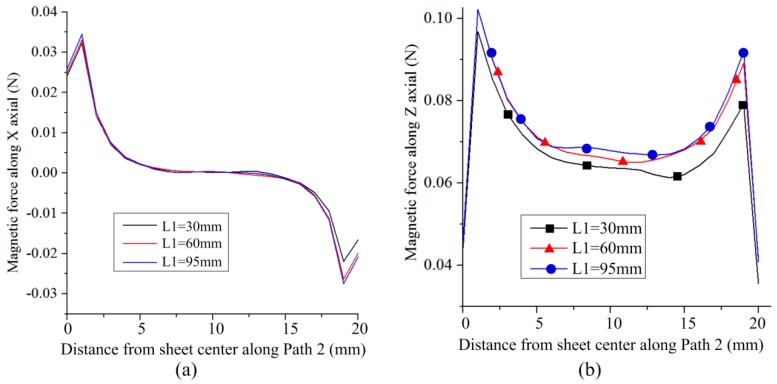
Effect of L1 on magnetic force along Path 2: (**a**) Along the X-axis and (**b**) along the Z-axis.

Figure 8 shows the distribution of magnetic force on Path 2 along the X-axis. Because the width of the sheet is smaller than the width of the left half of the coil, the two sides of the sheet were subjected to magnetic force along the X-axis. Thus, the sheet tends be compressed in the width direction. Figure 8b shows the distribution of magnetic force on Path 2 along the Z-axis. The larger the L1, the larger the magnetic force generated on Path 2 and the difference in magnetic force between the left and right sides of the sheet decreased. Thus, the uniformity of magnetic field force on the sheet was improved with the increase in L2.

### 4.3. Distance of Center Line between the Sheet and Left Section of Coil (L2)

**Figure 9:**
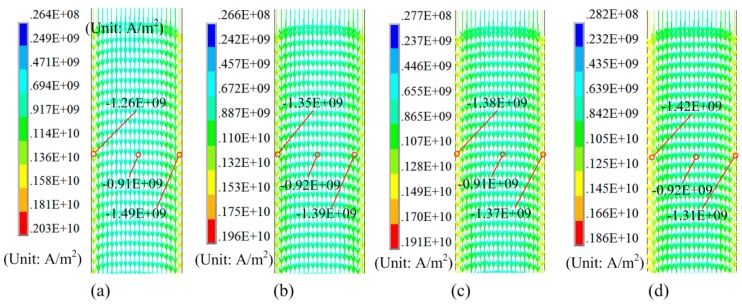
Effect of L2 on current density. (**a**) L2 = −2 mm, (**b**) L2 = −1 mm, (**c**) L2 = −0.7 mm, and (**d**) L2 = 0 mm.

Based on the results shown in Figure 7 and Figure 8, the magnetic force on the left side of the sheet is larger than that on the right side of the sheet, with a different value of L1. Therefore, it is necessary to appropriately move the sheet to offset the center line of the sheet from the left part of the coil. If L1 = 95 mm, L3 = 20 mm, and the resistivity of the die was set to 5e-7 Ω·m., Figure 9 shows the current distribution on the sheet with a different value for L2. The magnetic force on the left side of the sheet decreased, while the magnetic force on the right side of the sheet increased with the decrease in L2. The optimum value of L2 is −0.7 mm, corresponding to a nearly consistent current density on both sides of the sheet.

**Figure 10:**
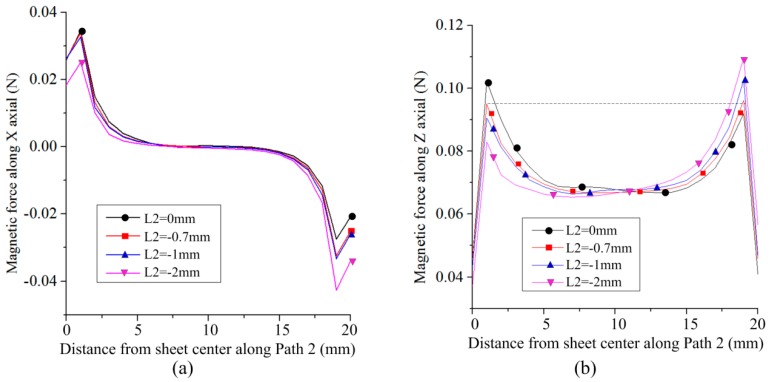
Magnetic force on the sheet after offset of the sheet from the coil: (**a**) Along the X-axis and (**b**) along the Z-axis.

Figure 10a shows the distribution of magnetic force on Path 2 along the X-axis. It could not significantly inhibit the magnetic force distribution along the X-axis on the sheet by appropriately moving the sheet. If L2 is equal to −0.7 mm, a magnetic force along the Z-axis occurs at both sides of the sheet.

### 4.4. Width of Sheet (L3)

**Figure 11:**
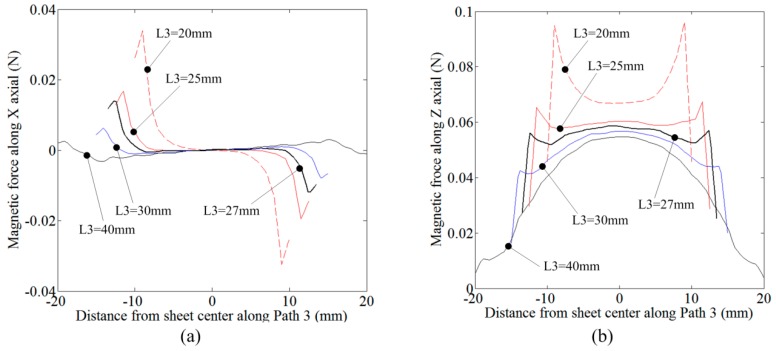
Effect of sheet width on magnetic force: (**a**) Along the X-axis and (**b**) along the Z-axis.

According to the results shown in Figure 10, the magnetic force on the sheet can be symmetrically distributed on the sheet by appropriately moving the sheet. However, the magnetic force on both sides of the sheet is much larger than that at the middle of the sheet if L3 = 20 mm. Therefore, the width of the sheet should be changed. Figure 11 shows the distribution of magnetic force on the sheet along the X- and Z-axes with different sheet widths. With the increase in sheet width, the magnetic force along the X-axis at both sheet sides significantly decreased. If the sheet width is equal to 20 mm and 25 mm, the magnetic force on Path 2 along the Z-axis shows a concave shape. If the sheet width is equal to 30 mm and 40 mm, the magnetic force on Path 2 along the Z-axis appears as a central protrusion shape. If L2 = 27 mm, which is equal to the length of the left part of the coil, the magnetic force on Path 2 along the Z-axis is distributed uniformly.

## 5. Manufactured Process and Magnetic Analysis for Coil

**Figure 12:**
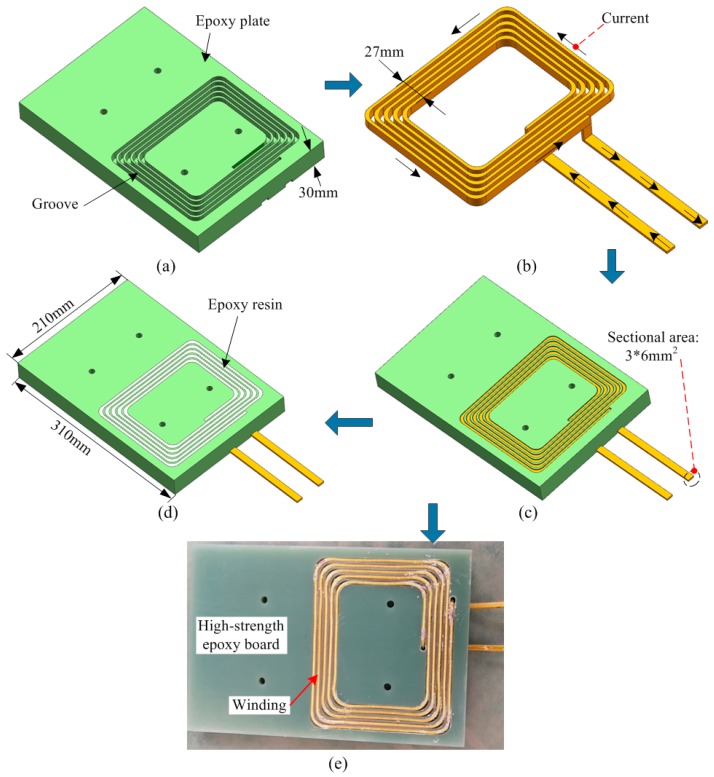
Manufacture process for a coil: (**a**) Make a groove in epoxy plate with CNC machining; (**b**) wire winding; (**c**) assemble; and (**d**) encapsulate with epoxy. (**e**) The actual coil before encapsulated.

Figure 12 shows the flow chart of manufacturing process for the flat spiral uniform pressure coil. First, a high-strength insulating material is selected as the supporting frame for the coil. Many insulating materials, such as nylon, bakelite, and epoxy plate, can be selected. In this paper, the epoxy plate was selected due to its high strength (bending strength greater than 340 MPa) and inexpensive price. According to the design parameters of the coil in Figure 2 and the simulation results in Chapter 4, the CNC machining was used to obtain the groove in epoxy plate, as shown in Figure 12a. Then, the 3*6 mm^2^ copper wires were manually winded according to the shape of the groove, as shown in Figure 12b. The shape of winding coil contains a spiral and two straight segments. The two straight segments were used to subsequently connect to the discharge device to form a current loop. Figure 12c shows the assembly result of the epoxy sheet and the winding wire. Since the winding wire was embedded in the epoxy board, there existed small gap. In order to avoid the shaking of the winding wire during the forming process, the assembled coil was encapsulated with epoxy resin. Thus, a working coil which can be used for the electromagnetic forming experiment was obtained, as shown in Figure 12d. Figure 12e is an actual diagram of the assembly result of the coil and the epoxy board.

**Figure 13:**
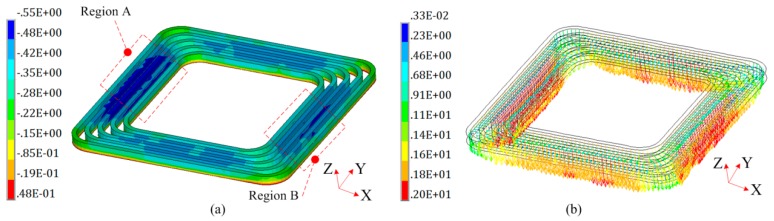
Magnetic force on the coil (Unit: N). (**a**) Legend; (**b**) vector diagram.

Under the condition that the sheet width is 30 mm, Figure 13 shows the distribution of magnetic force along the Z-axis on the flat spiral uniform pressure coil at 20 μs. It can be found that the direction of magnetic force acting on the winding along the Z-axis. Thus, the direction of the magnetic force in winding is opposite to the direction of sheet deformation. In order to more clearly describe the direction of the magnetic force on the winding, special regions, named A and B, were selected, as shown in Figure 13a.

**Figure 14:**
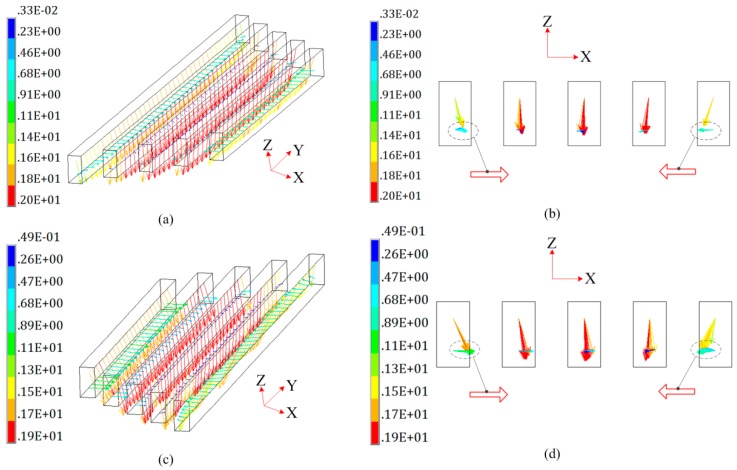
Magnetic force on special region (Unit: N). (**a**) Region A with 3D view; (**b**) region A with 2D view; (**c**) region B with 3D view; and (**d**) region B with 2D view.

In Figure 14, the regions A and region B of the coil are totally subjected to electromagnetic forces along the Z-axis. Both region A and region B contain five turns wires. The internal three turns wires are subjected to a large electromagnetic force along the Z-axis. The external two turns wires have a small Z-axis electromagnetic force. However, the external two turns wires are subjected to a certain magnetic force along the X-axis. Thus, the five turns wires of the region A and B have a tendency to be compressed. However, the electromagnetic force along the X-axis is much smaller compared to the one along the Z-axis, and the coil wire is packaged in a high-strength epoxy board which has a large resistance to compression. Therefore, the possibility of the coil failing in the X direction is relatively small.

**Figure 15:**
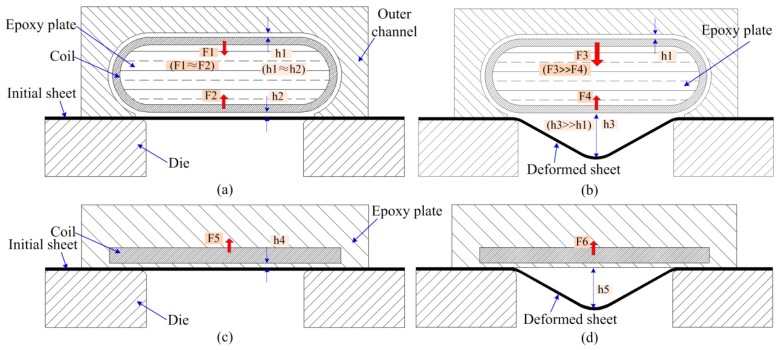
Deformation process under uniform magnetic pressure. (**a**) Initial condition in a conventional uniform pressure coil; (**b**) a deformed sheet in a conventional uniform pressure coil; (**c**) initial condition in a flat spiral uniform pressure coil; and (**d**) a deformed sheet in a flat spiral uniform pressure coil.

In order to further illustrate the advantages of the flat spiral uniform pressure coil proposed in this paper, the deformation process of the sheet by the conventional uniform pressure coil and the flat uniform pressure coil was analyzed, as shown in Figure 15. It can be seen that a die structure is required during the deformation of the sheet. The differences between the two uniform pressure coils are as follows: (1) The flat pressure coils require a flat spiral winding and an epoxy plate supporting frame; (2) the conventional uniform pressure coil needs to be winded with a 3D shape, and an epoxy plate supporting frame must be inserted in the winding wire. Moreover, it needs to manufacture a high conductivity external channel. It also needs to fill the region between metal outer channel and the 3D winding wire with insulating material to avoid contact. Therefore, the planar pressure coil proposed in this paper has a simple manufacturing process and a low manufacturing cost.

The deformation process of the sheet corresponding to the conventional uniform pressure coil is shown in Figure 15a,b. If the sheet has no deformation, the distance between the upper part of the coil and the outer channel is set to be h1, while the lower part of the coil and the sheet is set to h2. After the coil discharge, the upper part of the coil and the outer channel, as well as the lower part of the coil and the sheet will all generate a magnetic field concentration. As a result, the upper and lower parts of the coil are subjected to the magnetic force of F1 and F2, and the direction of the magnetic field force is away from the sheet and the outer channel, respectively. Thus, if h1≈h2, F1≈F2. However, the distance between the lower portion of the coil and the sheet (h3) is greatly increased if the sheet deforms. As a result, the strength of the magnetic field between the lower portion of the coil and the sheet material is greatly reduced, so the electromagnetic force (F4) obtained at the lower portion of the coil is greatly reduced. Since the distance between the upper part of the coil and the outer channel does not change, the upper part of the coil is still subjected to a large electromagnetic force F3. Thus, F3 > F4 after sheet deformation. Under this state of magnetic force, the upper portion of the coil will push the insulating material and cause the lower portion of the coil to bulge, which will cause the coil to fail, as shown in Figure 1d. Moreover, the conventional pressure coil has difficulty handling the failure of the coil.

The deformation process of the flat spiral pressure coil proposed in this paper is shown in Figure 15c,d. In the initial stage of sheet deformation, the coil wire is subjected to an electromagnetic force (F5) away from the direction of sheet deformation. If the sheet deforms, the distance between the sheet and the coil increases, and the electromagnetic force (F6) becomes smaller. Moreover, the direction of the magnetic field force is still away from the direction of the sheet deformation. While the coil is made, the coil wires are embedded in the grooves made of the high-strength epoxy board. The epoxy board has a large thickness and can fully withstand the reaction force along the Z-axis.

Therefore, the flat spiral pressure coil proposed in this paper can produce a uniform magnetic force distribution on the sheet, reduce the manufacturing difficulty, reduce manufacturing cost, and enhance the service life for the coil.

## 6. Sheet Deformation after Coil Discharge

**Figure 16:**
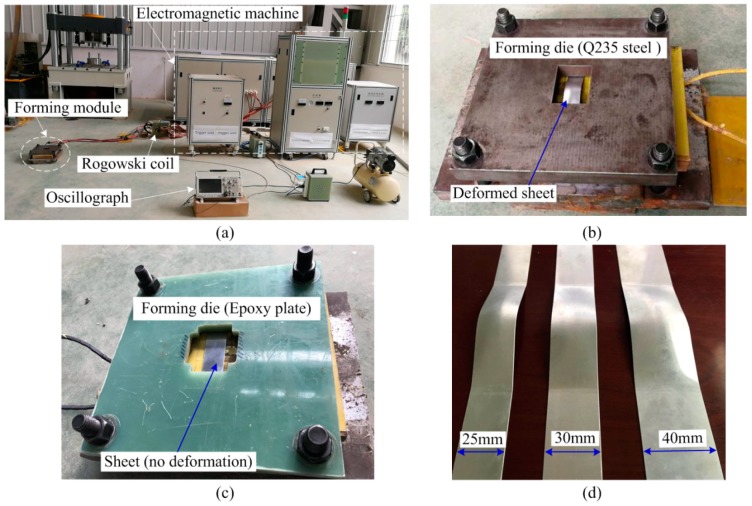
Experimental device and the deformed sheet. (**a**) 200 kJ EMF equipment; (**b**) steel die; (**c**) epoxy plate die; and (**d**) a deformed sheet with a different sheet width.

Figure 16 shows the experimental device and the deformed sheet at different discharge voltages. The EMF equipment was manufactured by Central South University. The main parameters are: The maximum discharge-energy (200 kJ), rated voltage (25 kV), and capacitance (640 μF). A Rogowski coil was used to measure the pulse current data passing through the working coil. An oscilloscope was used to record the current data. Finally, this current data were imported into numerical simulation software to calculate the magnetic force and dynamic forming process. Figure 16b shows a forming die made by Q235 steel, and the sheet is deformed after the coil is discharged. Figure 16c shows a forming die processed by an epoxy plate, and it can be seen that the sheet is not deformed after the discharge of the coil. This is because the lager and uniform induced the current generated on the sheet using the steel die. Figure 16d shows the final deformation results of the sheets at different sheet widths.

Electromagnetic forming involves the coupling of electromagnetic fields, structural fields, and temperature fields. If the coil discharges, a large current will be generated on the sheet, which may cause thermal softening. In the field of electrically assisted forming, Egea et al. [19] used electrically assisted (EA) wire drawing to enhance the formability, ductility, and elongation of the wire specimen. It is observed that electropulsing induces an ultrafast annealing process, which decreases the hardness in the center and surface of specimens. Valoppi et al. [20] used double-sided incremental forming with electrically-assisted forming to manufacture a difficult-to-form Ti6Al4V sheet. They found the formability and the geometric accuracy were increased, and the forming force was reduced. In addition, the joule effect prevented the surface defect from cracking. 

**Figure 17:**
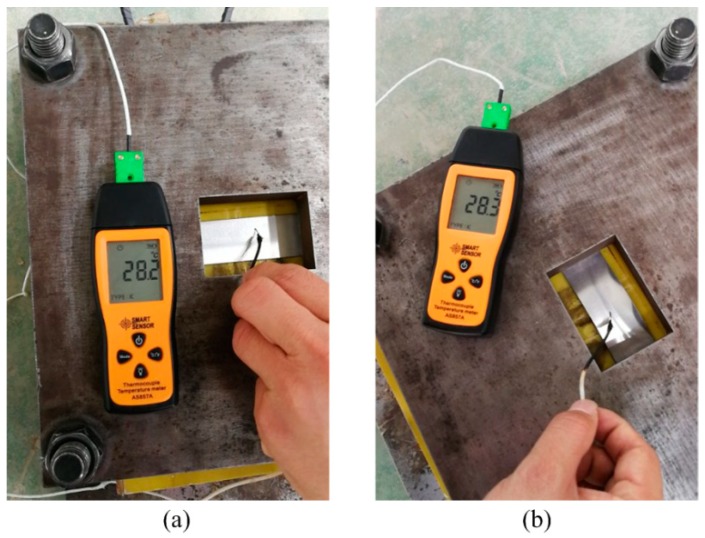
Temperature changes (**a**) before discharge and (**b**) after discharge.

However, the effects of temperature on electromagnetic forming are often ignored. This is because there is no significant temperature rise on the part, although the induced current was generated on the part after discharge. For example, Cui et al. [21] showed the tube had a temperature rise of about 5 °C after discharge based on a numerical simulation. In order to further explain why the temperature rise on the sheet during the electromagnetic forming process is small, a thermocouple sensor was used to measure temperature on the sheet. The error for the thermocouple sensor was ± 0.1 °C. The experiment found that the temperature on the sheet before and after discharge was almost unchanged, as shown in Figure 17.

**Figure 18:**
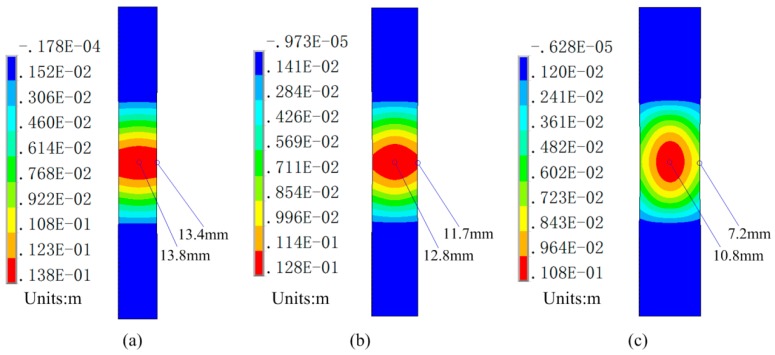
Deformed shape with different sheet widths: (**a**) L3 = 25 mm, (**b**) L3 = 30 mm, and (**c**) L3 = 40 mm.

Using the planar spiral uniform-pressure coil and by selecting the optimum technological parameters, Figure 18 shows the deformation results of sheet material under different sheet widths. The maximum displacement at the sheet center gradually increased with the decrease in sheet width. Moreover, the difference in displacement between the center of the sheet and the edge of the sheet decreased.

**Figure 19:**
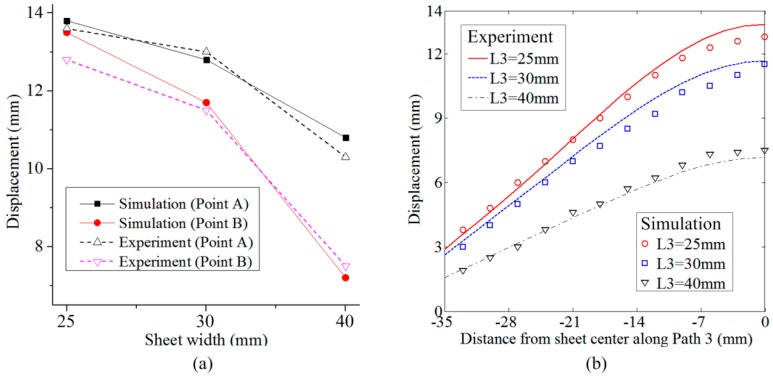
Simulation method verification: (**a**) A and B points and (**b**) sheet profiles.

Figure 19 shows the profiles on Path 3 and displacement at special points (A and B) obtained from the experiment and simulation with different sheet widths. The profiles between the experiment and simulation showed a slight deviation and small error at Points A and B. The maximum errors between experiment and simulation at A and B points were all less than 5.5%. Therefore, the simulation method has a high accuracy to predict electromagnetic sheet forming using a flat spiral uniform-pressure coil.

## 7. Conclusions

In this study, a new uniform-pressure coil with a planar structure was designed. The influencing factors on magnetic force distribution were analyzed by FEM. Some conclusions were obtained, as follows:

(1) A die with resistivity 5e-7 Ω·m and a spacing of 95mm between the left and right parts of the coil were used, and a current density larger than 1.31e9 A/m^2^ with the same flowing direction was generated on the sheet. By slightly moving the sheet, the magnetic forces were distributed symmetrically along the direction of the sheet deformation.
(2) The optimum sheet width was equal to the length of the left part of the coil, corresponding to the most uniform magnetic force along the Z-axis distribution on the sheet.
(3) Compared with the structure and magnetic force on a traditional uniform pressure coil, the flat uniform pressure coil proposed in this paper can produce a uniform magnetic force distribution on the sheet, reduce the manufacturing difficulty, reduce manufacturing cost, and enhance the service life for the coil.
(4) By comparing the experimental results, it was found that the simulation results have a higher analysis precision. The maximum errors between experiment and simulation at A and B points were all less than 5.5%.

## Figures and Tables

**Table 1:** Materials parameters of forming system.

**Sheet (5052 aluminum alloy)**	**Density (kg/m^3^)**	**2750**
	Elastic modulus (GPa)	68
	Yield strength (MPa)	105.8
	Electrical resistivity (Ω·m)	4.93 × 10^−8^
Coil (Copper)	Electrical resistivity (Ω·m)	1.7 × 10^−8^
Die (Q235 steel)	Electrical resistivity (Ω·m)	5 × 10^−7^
